# The multiple mediating effects of grit and learning agility on academic burnout and learning engagement among Korean university students: a cross-sectional study

**DOI:** 10.1080/07853890.2022.2122551

**Published:** 2022-10-03

**Authors:** Mi-Kyeong Jeon, Insook Lee, Mi-Young Lee

**Affiliations:** a Department of Nursing, Changwon National University, Changwon, Republic of Korea; b Nursing Department, College of Health and Welfare, Woosong University, Dong-gu, Daejeon, South Korea, Uijeongbu, Republic of Korea

**Keywords:** Academic burnout, learning engagement, grit, learning agility, mediating effect

## Abstract

**Introduction:**

This article reports the results of a study conducted to assess the mediating effects of grit and learning agility on the relationship between academic burnout and learning engagement among undergraduate students.

**Methods:**

A cross-sectional survey was conducted using a self-report questionnaire. Undergraduate students (*N* = 344) were recruited from one university in South Korea (58.0% female; average age 21.43) to complete assessments of academic burnout, grit, learning agility, and learning engagement. Data were analysed using descriptive statistics, Pearson’s correlation coefficient, hierarchical regression and bootstrapping to verify the multiple parallel mediation effect.

**Results:**

We found that the direct effect of academic burnout on learning engagement (B= −0.26, *p*<.001) and the indirect effect of academic burnout as mediated by learning agility (B= −0.13; 95% CI, −0.20∼−0.06) were significant. This finding confirmed that 33.3% of the total effect of academic burnout on learning engagement was the result of indirect effects *via* learning agility.

**Conclusion:**

These results indicate the necessity of developing an educational programme that focuses not only on reducing academic burnout but also on improving learning agility to increase undergraduate students’ learning engagement. This study contributes to the development of a curriculum aimed at increasing the effectiveness of university education, promoting learning engagement, and reducing academic burnout.

**KEY MESSAGE**
- Our study reports that academic burnout has both a direct effect on learning engagement and an indirect effect via learning agility. Learning agility mediates the relationship between academic burnout and learning engagement among undergraduate students.
- Although grit has been reported by many previous studies to mediate the relationship between the tendency to pursue happiness and the willingness to continue learning and effectively improving one’s academic achievement and ability, our study did not find any mediating effect via grit in this context.

## Introduction

Learning engagement is an important factor with respect to evaluating the quality of college education and improving the academic ability of undergraduate students [1]. Therefore, it is important to explore strategies for increasing the learning engagement of undergraduate students to improve their quality of learning. It has been suggested that learning engagement is a significant factor in improving learning satisfaction [1]. Flow can help individuals abandon self-consciousness, integrate the self [2], reduce burnout [3], and improve their sense of well-being [4]. On the other hand, contemporary society is unpredictable and is considered to have entered an era that is characterised by volatility, uncertainty, complexity, and ambiguity (VUCA) [5]. In these times, especially in Korea, as undergraduate students complete their studies, make career decisions, and prepare for employment, the primary goal of their learning is to enable them to enter the workforce. For this reason, undergraduate students may experience exhaustion due to long-term academic burnout [6]. More than 30% of college students complain of psychological distress and burnout symptoms resulting from extreme stress, and college students’ burnout is significantly correlated with suicidal thoughts and even suicide attempts, in severe cases [7]. Therefore, academic burnout is emerging as a global problem affecting undergraduate students, and it has a significant impact on students’ mental health and academic achievement [8,9].

Academic burnout can occur when students experience chronic academic stress for an extended period of time and fail to resolve it effectively [10]. Academic burnout has been commonly used as an indicator of learning engagement in previous studies [11–13]. Learning engagement has been linked to good academic performance [14,15]. As previously shown, lower levels of engagement are associated with elevated levels of depressive symptoms and burnout among medical students [16]. In contrast, grit is an essential factor with respect to controlling students’ academic burnout [17,18]. Grit is an essential characteristic for learners and predicts high achievement and low dropout rates in various fields, including education [19,20]. Moreover, grit has been found to have a significantly beneficial effect on performance after controlling for the effects of intelligence, experience, and talent in a specific field as well as demographic variables that affect individual achievement [19,20].

Learning agility has attracted attention as a critical talent requirement for the future of society [21], and talented people with high learning agility are highly likely to emerge as core talent [22,23]. People with excellent learning agility enjoy learning and growing by continually seeking new challenges, actively soliciting feedback from others, and reflecting on their experiences; thus, there is a strong possibility that practical results can be produced by studying these factors [21]. Additionally, people with high learning agility are likely to have a high degree of learning engagement. Previous studies have shown that the learning agility of corporate workers and teachers significantly influences their innovative work behaviour or workflow as a mediating or independent variable [24,25]. The learning agility model developed by DeRue and colleagues [26] suggests that contextual and environmental factors associated with individuals and organisations influence learning agility and that learning agility leads to positive outcomes. However, most previous studies have focussed on workers, and few studies have investigated university students. It is necessary to verify the relationship between college students’ learning agility and performance variables.

University education should focus on grit, which is an attribute of learners, and ways of increasing grit should be developed to reduce academic burnout and negative experiences among undergraduate students. Accordingly, the roles of grit and learning agility in the relationship between academic burnout and learning engagement require extensive investigation. Most previous studies focussing on college students’ learning engagement have been exploratory-level studies that have identified the variables that influencing learning engagement and subsequently analysed that influence. The ways in which these variables are related to students’ learning engagement remain unknown. It is particularly difficult to find studies that identify the variables that mediate the relationship between academic burnout and learning engagement. Grit and learning agility were chosen in this work as potential mediators of academic burnout and learning engagement, and these variables (grit and learning agility) can mediate the impact of academic burnout on learning engagement. Confirming the mediating effects of grit and learning agility on the relationship between academic burnout and learning engagement could facilitate the development of effective strategies to improve learning engagement by focussing on grit and learning agility.

### Aims

This study aimed to identify the levels of grit, learning agility, learning engagement, and academic burnout exhibited by undergraduate students and to examine the mediating effects of grit and learning agility on the impact of academic burnout on learning engagement. The specific research goals of this study were as follows: (1) to identify the relationships among undergraduate students’ academic burnout, grit, learning agility, and learning engagement; (2) to examine whether undergraduate students’ academic burnout affects their learning engagement; and (3) to determine whether grit and learning agility have multiple mediating effects on the relationship between academic burnout and learning engagement.

We hypothesised that students with higher grit and learning agility would exhibit higher levels of learning engagement. We also hypothesised that academic burnout affects learning engagement, whereas grit and learning agility mediate the impact of academic burnout on learning engagement.

## Methods

### Study design and setting

This study employed a cross-sectional survey to assess academic burnout, grit, learning agility and learning engagement among college students enrolled at a regional university in South Korea.

### Sampling and participants

The participants included undergraduate students enrolled at a regional university in South Korea, who were recruited between December 04 and 20, 2019. In Korea, nursing school admissions have remained highly competitive. The Korean government [27] has increased the quota for nursing department admissions by 30% to solve the problem of a shortage of nurses associated with the increased demand for nursing services resulting from the ageing population and changes in the healthcare environment. As a result, the nursing department is primarily selected as a major by undergraduates, although an increasing number of students in other majors are beginning to choose nursing as a second major due to the stability associated with nursing jobs. Therefore, to better improve our understanding of the current generation of nursing students, it is necessary to develop a better understanding of the characteristics of undergraduate students in general, accordingly, undergraduate students from regional universities were included as participants in this study.

The number of subjects was calculated using the G*Power 3.1.9.2 program (Faul, et al., Universitat Kiel, Germany). The F test for ANOVA was conducted with respect to seven groups, revealing a significance level (a) of 0.05, a power (1-ß) of 95%, and an effect size (f) of 0.25 (medium effect size). As a result, the required number of samples was calculated to be 343, and 350 people were sampled to account for dropout.

### Ethical considerations

This study was conducted after receiving approval from the IRB of Changwon National University (Approval number: 1040271-201910-HR-032). The IRB approved our study and deemed written informed consent to be unnecessary. Following approval by the IRB, we collected data after obtaining oral informed consent from the participants. We described the study to the students, explained the purpose of the study, noted that the participants could voluntarily cease participating in the study at any time and explained that there was no penalty associated with the choice not to participate. All participants provided informed oral consent and reviewed the explanatory documents associated with the study prior to participating. The participants completed the questionnaire anonymously and provided no personally identifying information. Individuals who voluntarily agreed to participate in the study were recruited.

### Conceptual framework

According to the learning agility conceptual framework developed by DeRue and colleagues [26], learning agility causes individual differences in the following three factors: goal orientation, metacognitive ability, and openness to experience. These factors constitute a basis for understanding an individual’s ability to learn from experience, and they affect learning agility *via* contextual and environmental factors. Learning agility promotes learning within and through experience, which leads to positive performance changes over time. Such agility in learning is an important factor that can predict an individual’s performance. In a study involving college students, learning agility was also found to be an important factor that affects career preparation behaviour *via* academic challenges and adaptation to college life. Thus, our study considers grit to be a contextual/environmental factor and assumes that learning agility is a cognitive/behavioural process that mediates academic burnout and learning engagement. If our study identifies the mediating effects of grit and learning agility on the relationship between academic burnout and learning engagement and identifies the mechanism by which academic burnout affects learning engagement, it can serve as a meaningful contribution to the task of developing a specific strategy for improving learning engagement. According to this study, grit and learning agility are predicted to reduce academic burnout in the context of university education.

### Measurement tools

The questionnaire consisted of two main sections. The first section included questions related to the demographic characteristics of participants, such as their age, gender, grade, academic field, interpersonal relationships, satisfaction with their major, grade point average (GPA), and reason for selecting their major. The second section collected data pertaining to participants’ academic burnout (Appendix 1), grit (Appendix 2), learning agility (Appendix 3), and learning engagement (Appendix 4).

#### Academic burnout

Academic burnout was measured using the Maslach Burnout Inventory-Student Survey (MBI-SS). This scale was initially developed by Schaufeli et al. [28] and was validated in the context of Korean university students by Shin et al. [29]. The survey is scored on a 5-point Likert scale and includes 15 questions, with higher scores indicating higher levels of academic burnout. The Cronbach’s α coefficients of the MBI-SS were measured as ranging from .65 to .86 by Schaufeli et al. [28], .82 to .86 by Shin et al. [29], and .87 in this study.

#### Grit

Grit was measured using the grit scale developed by Duckworth [30]. This scale includes 10 questions and is scored on a 7-point Likert scale. This scale includes questions regarding maintaining interest and continuing effort, and higher scores indicate higher levels of grit. The scale’s Cronbach’s α coefficient was measured as .85 by Duckworth et al. [19], .81 by Lim [31], and .74 in this study.

#### Learning agility

Learning agility was measured using a learning agility tool developed by Im et al. [21]. This tool uses a 7-point Likert scale to measure 5 factors (growth orientation, behaviour change, reflection pursuit, self-awareness, and flexible thinking) and includes a total of 22 questions; higher scores indicate higher levels of learning agility. The scale’s Cronbach’s α coefficients were measured as ranging from .86 to .91 by Im et al. [21] and as .89 in this study.

#### Learning engagement

Learning engagement was measured using a 29-item adult learner engagement scale developed and validated by Kim et al. [32]. This tool is scored on a 6-point Likert scale and includes 29 questions; higher scores indicate higher levels of learning engagement. The scale’s Cronbach’s α was measured as ranging from .65 to .90 by Kim et al. [32] and as .94 in this study.

### Data collection

This study proceeded with the data collection process after obtaining approval from the institutional review board (IRB) of the corresponding university. The data were collected between December 04 and 20, 2019. For recruitment, departments that allowed data collection were selected, and 30 to 70 paper questionnaires were distributed with the help of the departments’ offices following the approval of the study. It took approximately 15 to 20 min for each student to complete the questionnaire. The self-report questionnaires were completed using paper and pencil. In total, 350 questionnaires were distributed, and 344 questionnaires were completed (a response rate of 98.29%).

### Data analysis

We encoded the collected data, conducted = data analysis using Statistical Package for Social Sciences (SPSS) software for Windows (version 26.0; SPSS Inc., Chicago, IL, USA) and Hayes’s [33] PROCESS macro, ver. 3.4.1.

A frequency analysis of the subjects’ general characteristics was conducted, and descriptive statistics were used to analyse academic burnout, grit, learning agility, and learning engagement. Pearson’s correlation coefficient analysis was used to analyse the correlations among academic burnout, grit, learning agility, and learning engagement. A hierarchical regression analysis was conducted to confirm the mediating effects of grit and learning agility on the relationship between academic burnout and learning engagement. A multiple parallel mediation analysis was conducted using the PROCESS macro for SPSS ver. 3.4.1 as proposed by Hayes [33] specifically using Model 4. The significance of the mediating effect was analysed using the bootstrapping method with a 95% confidence interval.

The independence and normality of and equal variance in the residuals were determined to assess the assumptions of the regression analysis. First, the Durbin-Watson value was measured at 1.996 (approximately 2.00), indicating no autocorrelation of the error terms. A case-by-case diagnosis was performed to confirm the assumption of the normal distribution of the error term; the absolute value of the standardised residual was less than 3, the absolute value of Cook’s distance was less than 1, and the absolute value of the standardised difference in fit (DFFIT) was less than 2. Therefore, a normal distribution of the error terms could be assumed. Multicollinearity was tested, yielding a tolerance limit ranging from 0.12 to 0.89, and the variance inflation factor (VIF) of the variables ranged from 1.20 to 8.64. No multicollinearity problems were found with respect to the variables. Equal variance was tested using Breusch–Pagan’s test, indicating *p*>.05.

## Results

### General characteristics of participants

In total, 144 (42.0%) participants were male, and 199 (58.0%) participants were female; the participants’ average age was 21.43 (±1.94) years. In total, 73 (21.2%) participants were first-year students, 135 (39.2%) participants were second-year students, 69 (20.1%) participants were third-year students, and 67 (19.5%) participants were fourth-year students. Regarding their majors, 154 students (44.8%) majored in the humanities and social sciences, 100 students (29.1%) majored in the natural sciences, 22 students (6.4%) majored in the arts or physical fitness, and 68 students (19.8%) majored in engineering. Regarding the participants’ interpersonal relationships, 168 (49.4%) and 159 (46.8%) of the participants had strong and average interpersonal relationships, respectively. A total of 282 students (82.5%) were satisfied with their majors, and 203 (59.4%) students had GPAs in the moderate range (GPA 3.0 ∼ 3.9); 97 students (28.4%) chose their major based on employment considerations, while 89 students (26.0%) chose their major based on their aptitudes and interests (Table 1).

**Table 1.** General characteristics and learning agility, grit, academic burnout, and learning engagement scores of the participants (*N* = 344)^*^.

Variables	Categories	*n*(%)	AB		Grit		LA		LE	
			Mean ± SD	t/F (*p*)	Mean ± SD	t/F (*p*)	Mean ± SD	t/F (*p*)	Mean ± SD	t/F (*p*)
			2.64 ± 0.61		4.16 ± 0.70		5.00 ± 0.74		3.36 ± 0.71	
Gender	Male	144 (42.0)	2.50 ± 0.60	−3.77 (<.001)	4.35 ± 0.70	4.54 (<.001)	5.18 ± 0.78	3.85 (<.001)	3.46 ± 0.83	2.00 (.046)
	Female	199 (58.0)	2.75 ± 0.59		4.02 ± 0.66		4.87 ± 0.69		3.29 ± 0.61	
Age (yr)	18 ∼ 22	255 (74.8)	2.66 ± 0.59	0.78 (.437)	4.13 ± 0.68	−1.45 (.149)	5.00 ± 0.74	−0.09 (.930)	3.39 ± 0.70	1.01 (.314)
	23 ∼ 31	86 (25.2)	2.60 ± 0.66		4.25 ± 0.74		5.01 ± 0.74		3.30 ± 0.76	
Grade	First-year	73 (21.2)	2.58 ± 0.49	1.79 (.148)	4.20 ± 0.53	0.37 (.776)	5.00 ± 0.75	1.43 (.234)	3.46 ± 0.74	1.97 (.118)
	Second-year	135 (39.2)	2.71 ± 0.65		4.11 ± 0.73		5.09 ± 0.71		3.41 ± 0.68	
	Third-year	69 (20.1)	2.53 ± 0.55		4.20 ± 0.77		4.95 ± 0.80		3.20 ± 0.72	
	Fourth-year	67 (19.5)	2.70 ± 0.67		4.15 ± 0.73		4.88 ± 0.71		3.31 ± 0.73	
Academic field	Hhumanities and social sciences	154 (44.8)	2.72 ± 0.62	1.78 (.150)	4.14 ± 0.76	1.49 (.216)	4.99 ± 0.73	0.08 (.972)	3.34 ± 0.67	0.07 (.977)
	Natural science	100 (29.1)	2.60 ± 0.59		4.14 ± 0.61		5.01 ± 0.73		3.38 ± 0.64	
	Art and physical sciences	22 (6.4)	2.46 ± 0.65		4.46 ± 0.67		5.06 ± 0.74		3.37 ± 0.91	
	Engineering sciences	68 (19.8)	2.60 ± 0.59		4.12 ± 0.66		5.01 ± 0.78		3.37 ± 0.84	
Interpersonal relationships	Strong^a^	168 (49.4)	2.51 ± 0.62	10.59 (<.001) a < c	4.37 ± 0.67	24.02 (<.001) a,b > c	5.26 ± 0.71	24.24 (<.001) a > b,c	3.52 ± 0.75	8.88 (<.001) a = b=c
	Average^b^	159 (46.8)	2.75 ± 0.56		3.99 ± 0.60		4.80 ± 0.65		3.20 ± 0.63	
	Poor^c^	13 (3.8)	3.09 ± 0.62		3.34 ± 0.99		4.40 ± 0.88		3.20 ± 0.79	
Satisfaction with major	Satisfaction	282 (82.5)	2.53 ± 0.55	7.97 (<.001)	4.21 ± 0.66	−3.08 (.002)	5.05 ± 0.72	−2.12 (.034)	3.42 ± 0.69	−3.17 (.002)
	Not Satisfied	60 (17.5)	3.16 ± 0.59		3.91 ± 0.83		4.83 ± 0.81		3.10 ± 0.78	
GPA	High (≥4.0)^a^	95 (27.8)	2.51 ± 0.60	5.25 (.006) a < c	4.35 ± 0.68	6.81 (.001) a > c	5.14 ± 0.59	2.23 (.109)	3.53 ± 0.70	4.78 (.009) a > c
	Medium (3.0～3.9)^b^	203 (59.4)	2.65 ± 0.61		4.13 ± 0.68		4.97 ± 0.79		3.33 ± 0.72	
	Low (<3.0)^c^	44 (12.9)	2.86 ± 0.55		3.91 ± 0.73		4.91 ± 0.72		3.16 ± 0.70	
Reason for selecting their major	Employment^a^	97 (28.4)	2.80 ± 0.56	4.74 (<.001) a = b=c = d=e = f	4.02 ± 0.57	3.53 (.004) a = b=c = d=e = f	4.89 ± 0.67	3.10 (.009) a,b,c,d,f < e	3.24 ± 0.68	2.92 (.014) a = b=c = d=e = f
	Social popularity^b^	15 ( 4.4)	2.76 ± 0.76		3.91 ± 0.50		4.75 ± 0.89		3.29 ± 0.73	
	Recommendation^c^	54 (15.8)	2.75 ± 0.59		3.99 ± 0.70		4.85 ± 0.65		3.23 ± 0.70	
	Interest and aptitude^d^	89 (26.0)	2.45 ± 0.60		4.33 ± 0.73		5.09 ± 0.82		3.49 ± 0.74	
	Having a professional job^e^	51 (14.9)	2.47 ± 0.55		4.29 ± 0.74		5.28 ± 0.64		3.60 ± 0.67	
	Others (GPA, dreams since childhood)^f^	36 (10.5)	2.74 ± 0.63		4.27 ± 0.82		5.04 ± 0.76		3.25 ± 0.74	

^*^Missing data excluded. GPA: grade point average; AB: academic burnout; LA: learning agility; LE: learning engagement.

### Academic burnout, grit, learning agility, and learning engagement

A normal distribution was assumed because the skewness and kurtosis values of academic burnout, learning agility, grit, and learning engagement did not exceed the cut-off values. The average academic burnout score was 2.64 (±0.61), and the average grit score was 4.16 (±0.70). The average learning agility score was 5.00 (±0.74), and the average learning engagement score was 3.36 (±0.71) (Table 1).

### Differences in academic burnout, grit, learning agility, and learning engagement according to general characteristics

An analysis of differences in academic burnout, grit, learning agility, and learning engagement according to participants’ general characteristics (Table 1) revealed significant differences in academic burnout by gender (t=-3.77, *p*<.001), interpersonal relationships (*F* = 10.59, *p*<.001), satisfaction with one’s major (*t* = 7.97, *p*<.001), GPA (*F* = 5.25, *p*=.006), and reason for selecting one’s major (*F* = 4.74, *p*<.001). Grit was significantly higher in males than in females (t=-4.54, *p*<.001) as well as in subjects with strong and average interpersonal relationships (*F* = 24.02, *p*<.001), those who were satisfied with their major (t=-3.08, *p*=.002), and those with a GPA above 4.0 (*F* = 6.81, *p*=.001). Learning agility was significantly higher in males than in females (*t* = 3.85, *p*<.001) as well as in subjects with strong interpersonal relationships (*F* = 24.24, *p*<.001), those who were satisfied with their major (t=-2.12, *p*=.034), and those whose reason for selecting their major was to obtain a professional job (*F* = 3.10, *p*=.009). Learning engagement differed significantly according to gender (*t* = 2.00, *p*=.046), interpersonal relationships (*F* = 8.88, *p*<.001), satisfaction with one’s major (t=-3.17, *p*=.002), GPA (*F* = 4.78, *p*=.009), and reason for selecting one’s major (*F* = 2.92, *p*=.014).

### Correlations among academic burnout, grit, learning agility, and learning engagement

As shown by Pearson’s correlation coefficient analysis (Table 2), learning engagement was positively correlated with learning agility (*r*=.46, *p*<.001) and grit (*r*=.35, *p*<.001). In contrast, these variables were negatively correlated with academic burnout (r=-.37, *p*<.001). Academic burnout was negatively correlated with learning agility (r=-.32, *p*<.001) and grit (r=-.38, *p*<.001), and grit was positively correlated with learning agility (*r*=.41, *p*<.001).

**Table 2.** Correlations between learning agility, grit, academic burnout, and learning engagement (*N* = 344).

Variables	LA	Grit	AB	LE
	r (*p*)	r (*p*)	r (*p*)	r (*p*)
LA	1	.41 (<.001)	-.32 (<.001)	.46 (<.001)
Grit		1	-.38 (<.001)	.35 (<.001)
AB			1	-.37 (<.001)
LE				1

LA: learning agility; AB: academic burnout; LE: learning engagement.

### Multiple mediating effects of grit and learning agility on the relationship between academic burnout and learning engagement

The mediating effects were assessed by conducting a hierarchical multiple regression analysis that used four models to examine the explanatory power of the related independent variables that influenced learning engagement (Table 3).

**Table 3.** Factors influencing learning engagement (*N* = 344).

Variables	Model I		Model II		Model III		Model IV	
	B	t	B	t	B	t	B	t
Constants	3.01	13.20	4.15	12.68	3.29	8.12	1.93	4.33
Gender (0 = male)								
Female	−0.17	−2.15*	−0.07	−0.91	−0.01	−0.12	0.04	0.55
Interpersonal relationships (0 = poor)								
Strong	0.12	0.59	−0.03	0.17	−0.13	−0.63	−0.28	−1.43
Average	−0.16	−0.74	−0.19	−0.94	−0.30	−1.47	−0.35	−1.81
Satisfaction with major (0 = not satisfied)								
Satisfied	0.21	2.01*	0.02	0.17	0.02	0.15	0.04	0.37
GPA (0 = low)								
High	0.39	3.04**	0.28	2.24*	0.20	1.60	0.17	1.44
Moderate	0.15	1.25	0.09	0.75	0.06	0.52	0.08	0.72
Reason for selecting major (0 = employment)								
Social popularity	0.17	0.84	0.16	0.82	0.19	0.99	0.18	0.99
Recommendation	0.01	0.06	−0.04	−0.32	−0.04	−0.32	−0.03	−0.27
Interest and aptitude	0.17	1.62	0.08	0.83	0.06	0.56	0.06	0.61
Seeking a professional job	0.27	2.25*	0.20	1.73	0.18	1.53	0.11	1.03
Other (GPA or dream since childhood)	0.02	0.12	−0.01	−0.05	−0.07	−0.51	−0.10	−0.76
AB			−0.33	−4.72***	−0.28	−3.94***	−0.22	−3.30**
Grit					0.21	3.48**	0.13	2.16*
LA							0.32	6.02***
F Adj. R^2^ R^2^ change	4.346*** 0.099 0.129		6.101*** 0.154 0.056		6.757*** 0.183 0.030		9.548*** 0.263 0.080	

*p<.05, **p<.01, ***p<.001.

GPA: grade point average; AB: academic burnout; LA: learning agility.

The results of Model I showed that gender, satisfaction with one’s major, GPA, and reason for selecting one’s major significantly influenced learning engagement. Learning engagement was high among women (t=-2.15, *p*=.032), those who were satisfied with their major (*t* = 2.01, *p*=.045), those with a high GPA (*t* = 3.04, *p*=.003), and those who chose a major based on professional considerations (*t* = 2.225, *p*=.025). The explanatory power of the variables’ input in Model I was measured as 9.9% (*F* = 4.346, *p*<.001).

In Model II, GPA continued to have a significant effect on learning engagement, and academic burnout also had a significant influence. The results showed that participants with a high GPA (*t* = 2.24, *p*=.026) and those with low levels of academic burnout (t=-4.72, *p*<.001) exhibited high levels of learning engagement. The explanatory power of the variables added to Model II was 15.4% (*F* = 6.101, *p*<.001).

Model III showed that academic burnout and grit had significant influences on learning engagement. Lower levels of academic burnout (t=-3.94, *p*<.001) and higher levels of grit (*t* = 3.48, *p*=.001) were associated with increased learning engagement. The variable included in Model III explained 18.3% of the variance in learning engagement (*F* = 6.757, *p*<.001).

In Model IV, the general characteristic variables were not significant, but academic burnout, grit, and learning agility had significant influences. Lower levels of academic burnout (t=-3.30, *p*=.001), higher levels of grit (*t* = 2.16, *p*=.031), and higher levels of learning agility (*t* = 6.02, *p*<.001) were associated with greater learning engagement. The variables included in Model IV (*F* = 9.548, *p*<.001) explained 26.3% of the variance in learning engagement.

**Figure 1.**
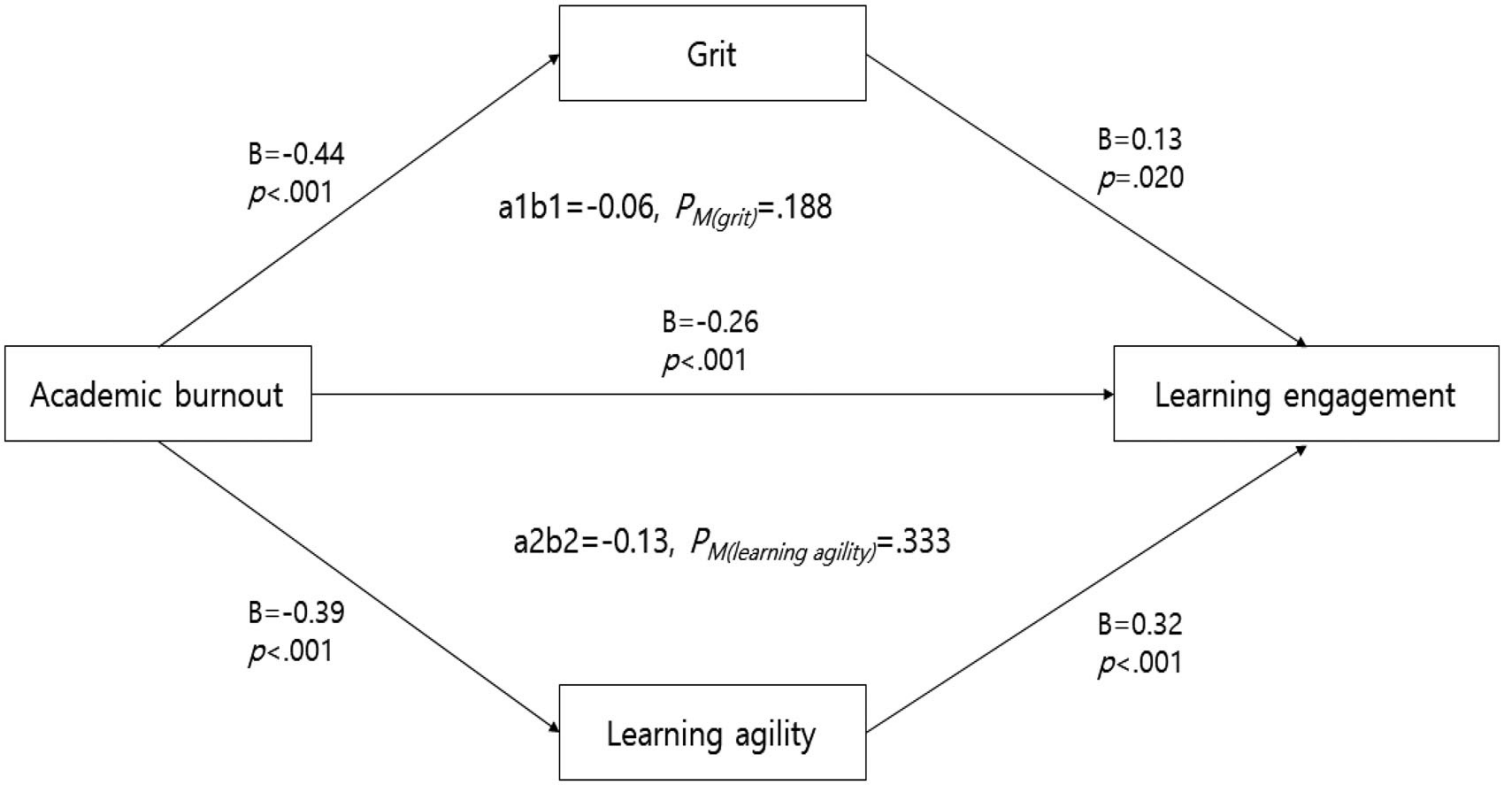
Multiple mediating effects of grit and learning agility on the relationship between academic burnout and learning engagement. Hayes’ PROCESS macro used. a1b1: indirect effect of academic burnout on learning engagement through grit; a2b2: indirect effect of academic burnout on learning engagement through learning agility; *P_M_*: ratio of indirect to total effect.

SPSS PROCESS macro Model 4 was used to construct a multiple parallel mediation model (Table 4, Figure 1), and bootstrapping was performed to verify the statistical significance of the mediating effect (Table 5). The indirect effect coefficient (B) of grit was measured as −0.06[−0.13, 0.01], which was not statistically significant at the 95% confidence level; however, the indirect effect coefficient (B) of learning agility was measured as −0.13[−0.20, −0.06], which was statistically significant at the 95% confidence level. The total mediating effect (B) was measured as −0.18[−0.29, −0.09] and was statistically significant at the 95% confidence level. Academic burnout was found to significantly affect learning engagement by as much as −0.13 when mediated by learning agility, and *P_M_*, the ratio of the indirect and total effects of learning agility, was measured as 0.333 (Figure 1). Thus, 33.3% of the total effect of academic burnout on learning engagement was indirect and mediated by learning agility.

**Table 4.** Mediating effect of grit and learning agility on the relationship between academic burnout and learning engagement (*N* = 344).

Variables	B	SE	t	*p* Value	95% CI	
					Boot LLCI	Boot ULCI
AB→LE	−0.26	0.06	−4.28	<.001	−0.373	−0.138
AB→Grit	−0.44	0.06	−7.63	<.001	−0.551	−0.325
AB→LA	−0.39	0.06	−6.23	<.001	−0.510	−0.265
Grit→LE	0.13	0.05	2.34	.020	0.020	0.233
LA→LE	0.32	0.05	6.48	<.001	0.226	0.423

CI: confidential interval; LLCI: the lower limit of B in the 95% confidential interval; ULCI: the upper limit of B in the 95% confidential interval; LA: learning agility; AB: academic burnout; LE: learning engagement.

**Table 5.** Direct and indirect effect on learning engagement by bootstrapping (*N* = 344).

Variables	Direct effect				Indirect effect			
	B	Boot SE	95% CI		B	Boot SE	95% CI	
			Boot LLCI	Boot ULCI			Boot LLCI	Boot ULCI
AB→LE	−0.26	0.06	−0.373	−0.138				
AB→Grit→LE					−0.06	0.03	−0.128	0.005
AB→LA→LE					−0.13	0.04	−0.203	−0.063
Total					−0.18	0.05	−0.286	−0.090

5,000 samples re-extracted for bootstrap; CI: confidential interval; LLCI: the lower limit of B in 95% confidential interval; ULCI: the upper limit of B in 95% confidential interval; AB: academic burnout; LE: learning engagement; LA: learning agility.

## Discussion

To solve the problems posed by shortage of nurses in the concrete situation of health care in Korea, the number of students in nursing colleges has increased, and as a result, nursing colleges have expanded significantly in terms of quantity. However, efforts to improve nursing students’ management of academic burnout and their learning engagement have been largely insufficient. In the past, diversification of the entrance examination environment strengthened the academic performance of nursing students, but there remains a lack of interest in learning agility, which has been shown to be a major variable associated with learning engagement and performance. This study was conducted to examine the multiple mediating effects of grit and learning agility on the relationship between academic burnout and learning engagement among undergraduate students. Based on the results of this study, we now have basic data concerning the management of academic burnout and the promotion of learning engagement among nursing students. We hypothesised that the higher individuals’ grit and learning agility are, the higher their learning engagement is; academic burnout affects learning engagement; and grit and learning agility mediate the impact of academic burden on learning engagement. Our study showed that students’ academic burnout affects their learning engagement and suggested that students’ learning agility significantly mediates the relationship between academic burnout and learning engagement.

### Academic burnout, grit, learning agility, and learning engagement

According to this study, the hypothesis that students with higher grit and learning agility exhibit higher levels of learning engagement was supported. The participants’ learning engagement was positively correlated with their learning agility and grit and negatively correlated with their academic burnout. Academic burnout was negatively correlated with learning agility and grit, whereas grit was positively correlated with learning agility. These results were consistent with those reported by Kim and Jang [17] and Park and Kang [34], who found that college students’ grit is significantly negatively correlated with academic burnout and that learning engagement is significantly negatively correlated with academic burnout.

Our study demonstrated that if interpersonal relations and satisfaction with one’s major improve, academic burnout can be reduced, and grit, learning agility, and learning engagement can be increased; in addition, this study found a positive correlation between learning engagement and learning agility. It is necessary to consider not only personal aspects but also social aspects and interpersonal relationships. Based on these results, college students’ learning engagement should be improved to mitigate their academic burnout. Nursing students experience a great deal of stress due to the heavy academic workloads, frequent tests, clinical practice and employment competition they face [35], and their levels of happiness are reported to be low due to this stress and these excessive academic burdens [36]. Among medical students, those in their second-year of education exhibit the highest rates of academic burnout [37], and undergraduates medical students react more cynically to their studies than students in graduate medical school, furthermore, the higher their rates of academic burden are, the more emotional exhaustion they experience in the context of academic burnout [38]. Accordingly, it can be seen that various educational strategies are necessary for these students, considering the fact that the academic burnout experienced by undergraduate medical students and students who receive training as healthcare providers, such as nursing and medical students, is higher than that encountered by other undergraduate students.

DeRue and colleagues [26] claimed that learning agility is affected by individual differences as well as by contextual and environmental factors and that it affects situational learning, thereby resulting in positive performance changes. Learning agility is a combination of basic cognitive skills; however, most importantly, it includes the motivation to think outside the box, to try new things and to learn from one’s experiences [26]. It is necessary to focus on learning agility as a determinant of college students’ ability to adapt, perform academically, and grow as social professionals. Moreover, in the case of nursing students, the development of learning agility, which refers to the ability to learn knowledge and apply it quickly in the rapidly changing context of healthcare, is also important. Various studies are necessary to identify the antecedents and outcomes of learning agility.

### Factors affecting learning engagement

Our hypothesis that academic burnout affects learning engagement was supported. The direct effect of academic burnout on learning engagement was significant. We expected that university students’ academic burnout would affect their learning engagement. The results of this study confirmed that academic burnout directly influences improvement in learning engagement. This finding was consistent with the conclusions of previous research suggesting that academic burnout is related to learning engagement [11–13]. However, learning engagement directly affects academic burnout and has an indirect effect on subjective well-being, as shown by a study focussing on college students in early childhood education departments [34]. These research findings revealed that learning engagement directly reduces academic burnout, thus contradicting the direction of the influencing factors. Students who apply deep-level learning processes experience less study-related burnout than students with other learning profiles [39]. The psychosocial stress experienced by medical students, according to an investigation of students in non-science majors, as well as the challenges to which this stress leads in both their academic and personal lives should have implications for helping all students by focussing on the ways in which they should be supported and the possibilities for developing curricula that can provide quality learning to these medical students [40]. Given current knowledge, however, counselling can be provided in a more focussed manner from the very beginning of a student’s academic career, thus facilitating specific educational or even therapeutic interventions. The literature has shown that the unorganised learning encountered by medical students causes academic burdens and pressure throughout the learning process accordance with a predetermined timetable [41]. Early dedicated counselling can help prevent or mitigate planning-related learning problems and, as a consequence, improve study success among medical students [42]. Therefore, further study is necessary to identify the different variables that affect undergraduate students’ academic burnout and to develop early interventions, such as the provision of counselling to support and manage students facing high levels of academic burnout. Additional research is needed to verify the relationship between academic burnout and learning engagement, as empirical studies investigating the influence of academic burnout and learning engagement have not been conducted in Korea.

### Multiple mediating effects on the relationship between academic burnout and learning engagement

Our hypothesis that grit and learning agility mediate the impact of academic burnout on learning engagement was partially supported, as our results indicated that grit does not have a significant indirect effect on learning engagement; however, learning agility does have a significant indirect effect on learning engagement. Our results confirmed that academic burnout had both a direct effect on learning engagement and an indirect effect *via* learning agility. However, we confirmed that the indirect effect of grit was not significant. This result is inconsistent with previous findings suggesting that grit has a significant mediating effect. Grit was shown to have a positive effect on social competence and to mediate the relationship between happiness orientation and social competence fully in a study involving 380 Chinese university students [43]. Grit was found to mediate the relationship between the tendency to pursue happiness and the willingness to continue learning [44] as well as the relationship between having a growth mindset and academic participation [45]. Moreover, possession of patience and passion, the attributes of grit, and increased engagement partially predicted higher GPAs [47]. Some scholars have noted that the importance of grit is somewhat exaggerated because previous results concerning the relationship between grit and performance have been inconsistent [31,46].

This study did not find grit to have a mediating effect in this context, but previous studies have reported that grit effectively improves academic achievement and maintenance. Duckworth [30] noted that finding work that is of interest to the individual is the highest priority with respect to the development of grit and that interest can grow into passion when individuals autonomously decide what they like and continue to pursue certain activities. Grit has also been viewed as virtually unchanging and consistent in adulthood [47]. Therefore, it is necessary to offer college students experiences that involve various activities so that they can develop grit before this trait becomes fixed in adulthood. It is necessary to establish educational goals and develop a curriculum that can consistently reach these goals to improve the education of nursing students. The support of trusted colleagues and families who can encourage the development of grit is also associated with high levels of this trait [48]. Therefore, it is necessary to create and sustain support groups, such as mentoring programs with colleagues, seniors and juniors, and professors so that nursing students can share their difficulties and receive help throughout the learning process.

This study found that learning agility significantly mediates the relationship between academic burnout and learning engagement. Most studies related to learning agility have emphasised workers, and few studies have focussed on college students. College students’ learning agility fully mediates the relationship between their acceptance of diversity and career decisions [49]. Additionally, college students’ novel experiences, curiosity, degree of questioning, and other traits affect their learning agility [50] and thus have significant effects on adjustment to university life [51]. Learning agility mediates the relationship between informal learning and professional identity [25]. People who learn well are thought to adopt an open perspective concerning others and to be willing to control their behaviour in accordance with their environment by recognising cultural differences [52]. Therefore, learning agility can be considered to be an essential factor that significantly affects individual achievement and performance. Additionally, learning agility is thought to mediate the relationship between academic burnout and learning engagement significantly.

Research on academic burnout or learning engagement in nursing students or medical students has rarely been conducted, thus making it difficult to draw a generalised conclusion in this context; however, the academic burnout that nursing students experience during the learning process can hinder their health and well-being and can negatively affect their performance when working as new nurses in the nursing field [53]. In addition, since academic burnout is a gradual phenomenon, educational intervention is needed before it becomes serious, and preventive intervention should be offered to medical students facing severe academic stress [54]. Therefore, it is necessary to understand students’ experiences of academic burnout during their undergraduate years and to identify the factors that can buffer academic burnout. In essence, American medical students described that they were better able to focus on the task at hand and to explore learning opportunities more fully when they felt a sense of psychological safety [55]. Therefore, by providing a psychologically safe learning environment, learners’ learning engagement can be improved.

Meanwhile, discussions are emerging concerning the possibility of developing learning agility that emphasise ways of systematically developing and promoting learning agility from the perspective of human resource development [56]. Therefore, it is necessary to develop a plan to increase learning agility among university students as future members of society. Eichinger et al. [57] noted that people with high levels of learning agility experience and manage ambiguous and complex situations and challenge limits when they encounter new challenges and adopting new methods. It can be inferred that agility can be acquired by accumulating a variety of experiences. Therefore, it is possible to enhance learning engagement by way of learning agility by allowing college students to experience a variety of situations. However, since excessively challenging and complex problems can hinder individual learning [58], additional evidence is needed regarding the specific experiences that result in learning agility. Furthermore, it is difficult for individuals to engage in agile learning and develop a sense of psychological stability in organisations with narrowly defined goals and a culture of punishment; therefore, a culture that is characterised by tolerance for mistakes made during the learning process is essential [22,26]. Therefore, it is necessary to create a culture that allows for mistakes or failures while increasing opportunities to learn about the university’s culture by providing students with various experiences, achievements and challenges. It is also necessary to create an educational climate in which the characteristics of learning agility are exhibited when exploring new areas, learning resources are available, and newly learned content is applied flexibly [56].

### Limitations and strengths

Since this study was conducted at only one university in a single region, the generalisability of our findings may be limited by the convenience sampling methods we used. The general population of undergraduate students in Korea might be less sensitive to academic burnout and may thus have been unable to reflect on learning agility and learning engagement. Additionally, this research does not consider students’ majors and thus faces certain limitations. In particular, the nursing students of only one university were included in this study, which may have failed to reflect the characteristics of nursing students in general.

Despite these limitations, however, we believe that this study was able to identify the effects of academic burnout, grit, and learning agility on learning engagement in undergraduate students. Notably, our findings yielded rich insights into the factors influencing academic burnout and learning engagement among Korean undergraduate students. This study is meaningful because it confirmed the role of learning agility as an important variable in reducing academic burnout and increasing learning engagement throughout the learning processes of undergraduate students. However, future studies are necessary to explore academic burnout and learning engagement in other contexts. Specifically, research is needed to compare nursing students with other college students, to develop and evaluate the effectiveness of programs intended to manage academic burnout and improve learning engagement, and to explore this phenomenon on a national scale by conducting research at multiple institutions.

## Conclusion

This study confirmed the mediating effects of grit and learning agility on the relationship between academic burnout and learning engagement among college students. As a result, the empirical data confirmed that learning agility mediates the relationship between academic burnout and learning engagement. Based on these results, it is necessary in the future to develop various educational strategies to improve the learning agility of undergraduate students, who represent an important human resource for university education. To improve learning agility, it is necessary to increase students’ ability to develop learning agility by providing them with various experiences in the university curriculum. In future studies, qualitative research is necessary to explore the ways in which undergraduate students’ learning agility can be developed. Additionally, examining the relationships among academic burnout, grit, and learning agility in college students as well as the academic environment factors that affect learning engagement could allow us to make additional contributions to the development of educational programmes to improve learning agility and learning engagement.

## Data Availability

The data used and/or analysed in the context of the current study are available from the corresponding author upon reasonable request.

## Appendix 1. Maslach Burnout Inventory-Student Survey Scale (MBI-SS)

**Table:**

I feel emotionally drained by my studies. I feel used up at the end of a day at university. I feel tired when I get up in the morning and I have to face another day at the university. Studying or attending a class is really a strain for me. I feel burned out from my studies. I have become less interested in my studies since my enrolment at the university. I have become less enthusiastic about my studies. I have become more cynical about the potential usefulness of my studies. I doubt the significance of my studies. I can effectively solve the problems that arise in my studies. I believe that I make an effective contribution to the classes that I attend. In my opinion, I am a good student. I feel stimulated when I achieve my study goals. I have learned many interesting things during the course of my studies. During class I feel confident that I am effective in getting things done.

## Appendix 2. Original Grit Scale (Grit-O)

**Table:**

New ideas and projects sometimes distract me from previous ones. Setbacks don’t discourage me. I don’t give up easily. I often set a goal but later choose to pursue a different one. I am a hard worker. I have difficulty maintaining my focus on projects that take more than a few months to complete. I finish whatever I begin. My interests change from year to year. I am diligent. I never give up. I have been obsessed with a certain idea or project for a short time but later lost interest. I have overcome setbacks to conquer an important challenge.

## Appendix 3. Korean Learning Agility Scale

**Table:**

I know my own strengths and weaknesses clearly. I am well aware of my own emotional state. I know what influenced my own feelings and emotions. I believe that I can improve my own potential through effort. I am interested in growing to a higher level than now. I have a strong motivation to develop new skills. I am very interested in developing my career. I take other people’s feedback as an opportunity for growth. I draw a picture of myself and results in the future. I pursue a high level of goal. I present a new perspective by integrating different concepts and ideas. I also think about the invisible side of events or situations I think and approach problems or opportunities in a new way. I actively ask for feedback from other people about my learning activities. I constantly ask why and how about learning or activities that I am currently doing. I constantly explore the root causes of success and failure. I can explain the reasons before making decisions and actions. I'm willing to get out of my own comfort zone and try something new. I am not swayed by resistance to change. I am willing to take responsibility for the task of change. I do not fear failure and perceive it as part of innovation or learning process. I don’t hesitate to take risks even in uncertain situations.

## Appendix 4. Korean Learning Flow Scale for Adults

**Table:**

My ability is enough to fully understand the new content. It’s hard to study new content but I believe I can do it with my skills. I can challenge new learning content. I set the amount to study before I start studying. I clearly set a goal when I study. I know exactly what I have to do when I study. I feel like I am studying properly. I know exactly how well I'm doing while I'm studying. I can tell how well I am doing when I see what I am studying. When I study, it proceeds by itself without special thought. The process of studying is as natural as flowing water. I feel like the process of studying happens automatically. I tend to fall in love when I study. I don’t think about anything else when I study. I concentrate completely when I study. I feel like I have full control over myself when I study. I can do as I want when I study. I feel all the procedures are under my own wing when I study. I used to concentrate on studying to the extent that I forgot myself. When I study, I concentrate so much that I can’t understand even if other people talk. I don’t know what happens around me when I study. When I concentrate on studying, the speed of time feels different from usual. Time flies while I'm studying. Time flies so fast while I'm studying. I enjoy studying regardless of the result. I feel happy while studying. The process of studying itself is fun. I enjoy studying time. Studying is exciting for me.

## Abbreviations

DFFIT: difference in fit
GPA: grade point average
IRB: institutional review board
MBI-SS: Maslach Burnout Inventory-Student Survey
SPSS: Statistical Package for Social Science
VIF: variance inflation factor
VUCA: volatility, uncertainty, complexity, and ambiguity
